# RETRACTION: SIRT5‐Related Desuccinylation Modification Contributes to Quercetin‐Induced Protection against Heart Failure and High Glucose‐Prompted Cardiomyocytes Injured through Regulation of Mitochondrial Quality Surveillance

**DOI:** 10.1155/omcl/9805267

**Published:** 2026-08-06

**Authors:** Oxidative Medicine and Cellular Longevity

RETRACTION: X. Chang, T. Zhang, J. Wang, et al., “SIRT5‐Related Desuccinylation Modification Contributes to Quercetin‐Induced Protection against Heart Failure and High Glucose‐Prompted Cardiomyocytes Injured through Regulation of Mitochondrial Quality Surveillance,” *Oxidative Medicine and Cellular Longevity*, vol. 2021 (2021). https://doi.org/10.1155/2021/5876841.

The above article, published online on 24 September 2021 in Wiley Online Library (wileyonlinelibrary.com), has been retracted following the agreement of the Journal’s Chief Editor Jeanette Vasquez‐Vivar; and John Wiley & Sons Ltd.

This article has been retracted following an investigation undertaken by the publisher. This investigation has uncovered evidence of one or more of the following indicators of systematic manipulation of the publication process:

1. Discrepancies in scope
2. Discrepancies in the description of the research reported
3. Discrepancies between the availability of data and the research described
4. Inappropriate citations
5. Incoherent, meaningless and/or irrelevant content included in the article
6. Manipulated or compromised peer review

The presence of these indicators undermines our confidence in the integrity of the article’s content and we cannot, therefore, vouch for its reliability. Please note that this notice is intended solely to alert readers that the content of this article is unreliable. We have not investigated whether authors were aware of or involved in the systematic manipulation of the publication process.

The authors were informed of this retraction but did not provide a response.

